# Both gastrocnemius aponeurosis flaps and semitendinosus tendon grafts are effective in the treatment of chronic Achilles tendon ruptures – a systematic review

**DOI:** 10.1186/s12891-023-07064-8

**Published:** 2023-12-08

**Authors:** Niklas Nilsson, Immanuel Stensöta, Katarina Nilsson Helander, Annelie Brorsson, Michael R. Carmont, Sebastian Concaro

**Affiliations:** 1 Department of Orthopaedics, Institute of Clinical Sciences at Sahlgrenska Academy, University of Gothenburg, Gothenburg, Sweden; 2 Department of Orthopaedics, Sahlgrenska University Hospital, Göteborgsvägen 31, Mölndal, 431 80 Sweden; 3 IFK Kliniken Rehab, Gothenburg, Sweden; 4 Department of Orthopaedic Surgery, Princess Royal Hospital, Shrewsbury and Telford Hospital NHS Trust, Shropshire, UK

**Keywords:** Chronic Achilles tendon rupture, Semitendinosus tendon graft, Gastrocnemius aponeurosis flap, Surgical repair, Systematic review, MINORS

## Abstract

**Introduction:**

A chronic Achilles tendon rupture (ATR) is defined as an ATR that has been left untreated for more than four weeks following rupture. This systematic review aims to summarize the outcomes of chronic ATR treated using either a gastrocnemius aponeurosis flap or semitendinosus tendon graft.

**Methods:**

A systematic search was conducted in three databases (PubMed, Scopus and Cochrane), for studies describing outcomes after surgical treatment of chronic ATR using gastrocnemius aponeurosis flaps or semitendinosus tendon grafts with more than 10 patients included. The studies were assessed for quality and risk of bias using the Methodological Items used to assess risk of bias in Non-Randomized Studies (MINORS).

**Results:**

Out of the 818 studies identified with the initial search, a total of 36 studies with 763 individual patients were included in this systematic review. Gastrocnemius aponeurosis flap was used in 21 and semitendinosus tendon graft was used in 13 of the studies. The mean (SD) postoperative Achilles tendon Total Rupture Score (ATRS) for patients treated with a gastrocnemius aponeurosis flap was 83 (14) points and the mean (SD) American Orthopaedic Foot and Ankle Score (AOFAS) was 96 (1.7) points compared with ATRS 88 (6.9) points and AOFAS 92 (5.6) points for patients treated with a semitendinosus tendon graft. The included studies generally had low-quality according to MINORS, with a median of 8 (range 2–13) for all studies.

**Conclusion:**

Both gastrocnemius aponeurosis flaps and semitendinosus tendon grafts give acceptable results with minimal complications and are valid methods for treating chronic ATR. The main difference is more wound healing complications in patients treated with a gastrocnemius aponeurosis flap and more sural nerve injuries in patients treated with a semitendinosus grafts. The current literature on the subject is of mainly low quality and the absence of a patient-related outcome measure validated for chronic ATR makes comparisons between studies difficult.

**Level of evidence:**

Level IV.

## Introduction

Medical history and clinical examinations are considered to be sufficient in establishing the diagnosis of an acute Achilles tendon rupture [34, 42]. However, occasionally treatment is delayed due to late presentation or misdiagnosis [8, 42]. A delay of treatment leads to larger tendon-end diastasis with interposed scar tissue [9]. Achilles tendon ruptures that have had a delay in treatment by more than 4 weeks, are termed chronic and surgical intervention is required to recover lower leg function [1, 7, 14, 28, 30]. The traditional surgical intervention for chronic Achilles tendon ruptures involves considerably larger incisions than acute repairs leading to an increased risk of complications, such as infections and inadequate wound-healing [41].

Patients with chronic Achilles tendon ruptures report different symptoms compared with those of acute ruptures. Long-term pain and recurrent swelling are more frequent in patients with chronic ruptures [28]. In addition, altered gait with a weakness at push-off, a poor balance, and a reduced capability of performing heel-rises are commonly reported [17, 42].

The literature includes various surgical techniques for the management of chronic Achilles tendon ruptures: V–Y plasty, tendon transfers, gastrocnemius aponeurosis/fascia flaps, direct repair and synthetic grafts, [1, 8, 14, 27, 28, 30, 35, 47, 66, 68]. In recent years endoscopically assisted techniques have been advocated by the literature, including endoscopic transfers of the FHL-tendon [16], peroneus brevis tendon [39] and semitendinosus tendon [46] due to the lower risk of skin complications and wound infections. The choice of surgical procedure depends on the location of the rupture, the size of tendon-end diastasis, individual factors such as patient activity level and age, together with the preference and experience of the surgeon [51].

Even though many surgical techniques are described in the literature, no single technique has been shown to be superior to another. The aim of this systematic review was to analyse the current clinical evidence of two established techniques to treat chronic Achilles tendon ruptures: gastrocnemius aponeurosis flaps and semitendinosus tendon grafts as both FHL-grafts [2] and local tendon transfers [43] have been discussed in recent reviews. However, due to a high heterogenicity between the included studies, it was not deemed possible to do any statistical comparing between the two techniques.

## Method and materials

### Search query

The systematic search was performed on 2021–07-02, with an updated search a year later, on 2022–09-22, in three established databases: PubMed, Scopus and Cochrane Library using the search queries outlined in Table 1. The initial search query included all studies presenting the outcome of chronic Achilles tendon ruptures treated surgically. In the final stage, studies presenting outcomes of gastrocnemius aponeurosis flaps or semitendinosus tendon grafts were isolated and analysed. The inclusion criteria were all studies, descriptive and comparative, presenting results on the management of chronic Achilles tendon ruptures in adults (> 18 years) using gastrocnemius aponeurosis flap or semitendinosus tendon graft. Numerous studies also included patients surgically treated for a re-rupture using the same technique. The data on re-ruptures was excluded when presented separately. In those studies where it was not possible to separate the results, the study was still included if a majority of the included patients were treated for a chronic Achilles tendon rupture. Other exclusion criteria were (1) case-reports and case series with less than 10 patients, (2) reviews, (3) studies written in non-English languages and (4) expert opinions. The systematic review was registered in PROSPERO (CRD42022294130).

**Table 1:** Search query, Boolean operators and search results in the databases PubMed, Scopus, and Cochrane

PubMed	Search string	Limits	Number of records
#1	(Achilles Tendon[mh] OR “Achilles tendon” [tiab] OR “Achilles tendons”[tiab] OR “Calcaneal Tendon”[tiab] OR “Calcaneal Tendons”[tiab] OR “Tendo calcaneus”[tiab])		12 515
#2	(chronic*[tiab] OR neglect*[tiab] OR miss*[tiab] OR delay*[tiab])		2 204 598
#3	#1 AND #2		1 750
#4	(Rupture[mh] OR rupture[tiab] OR ruptures[tiab] OR tear*[tiab])		192 888
#5	(Treatment Outcome[mh] OR treatment[tiab] OR therapy[tiab] OR outcome*[tiab] OR “clinical efficacy” [tiab] OR “clinical effectiveness” [tiab] OR complication*[tiab] OR re-rupture*[tiab] OR rerupture*[tiab])		8 235 689
#6	#3 AND #4 AND #5		526
Scopus	Search string	Limits	Number of records
#1	TITLE-ABS-KEY ("Achilles tendon" OR "Achilles tendons" OR "Calcaneal Tendon" OR "Calcaneal Tendons" OR "Tendo calcaneus")		16 771
#2	TITLE-ABS-KEY (chronic* OR neglect* OR miss* OR delay*)		4 433 517
#3	#1 AND #2		2 439
#4	TITLE-ABS-KEY (rupture OR ruptures OR tear*)		387 825
#5	TITLE-ABS-KEY (treatment OR therapy OR outcome* OR “clinical efficacy” OR “clinical effectiveness” OR complication* OR re-rupture* OR rerupture*)		14 409 980
#6	#3 AND #4 AND #5		803
#7	#3 AND #4 AND #5	Limit to: Article, Review	762
Cochrane	Search string	Limits	Number of records
#1	("Achilles tendon" OR "Achilles tendons" OR "Calcaneal Tendon" OR "Calcaneal Tendons" OR "Tendo calcaneus"):ti,ab,kw		1004
#2	(chronic* OR neglect* OR miss* OR delay*):ti,ab,kw		232 193
#3	#1 AND #2		206
#4	(Rupture OR ruptures OR tear*):ti,ab,kw		13 308
#5	(Treatment OR therapy OR outcome* OR “clinical efficacy” OR “clinical effectiveness” OR complication* OR re-rupture* OR rerupture*):ti,ab,kw		1 344 590
#6	#3 AND #4 AND #5		31
#7	#3 AND #4 AND #5	Limit to: Trials	30

### Study selection

The search was conducted by the authors and the initial search resulted in 1,340 studies, after removing duplicates, 818 studies remained. All studies were uploaded to the website Rayyan® for abstract review. The Preferred Reporting Items for Systematic reviews and Meta-Analyses (PRISMA) was used to structure the filtering of studies [54]. Two authors (NN and IS) independently reviewed the abstracts of the included studies. Disagreement between the authors were settled through discussion. A total of 182 studies remained after the initial abstract review. These were all later successfully imported as full-text versions. For the full-text review, the studies were divided equally between the six authors and checked by a minimum of two authors.

**Fig. 1:**
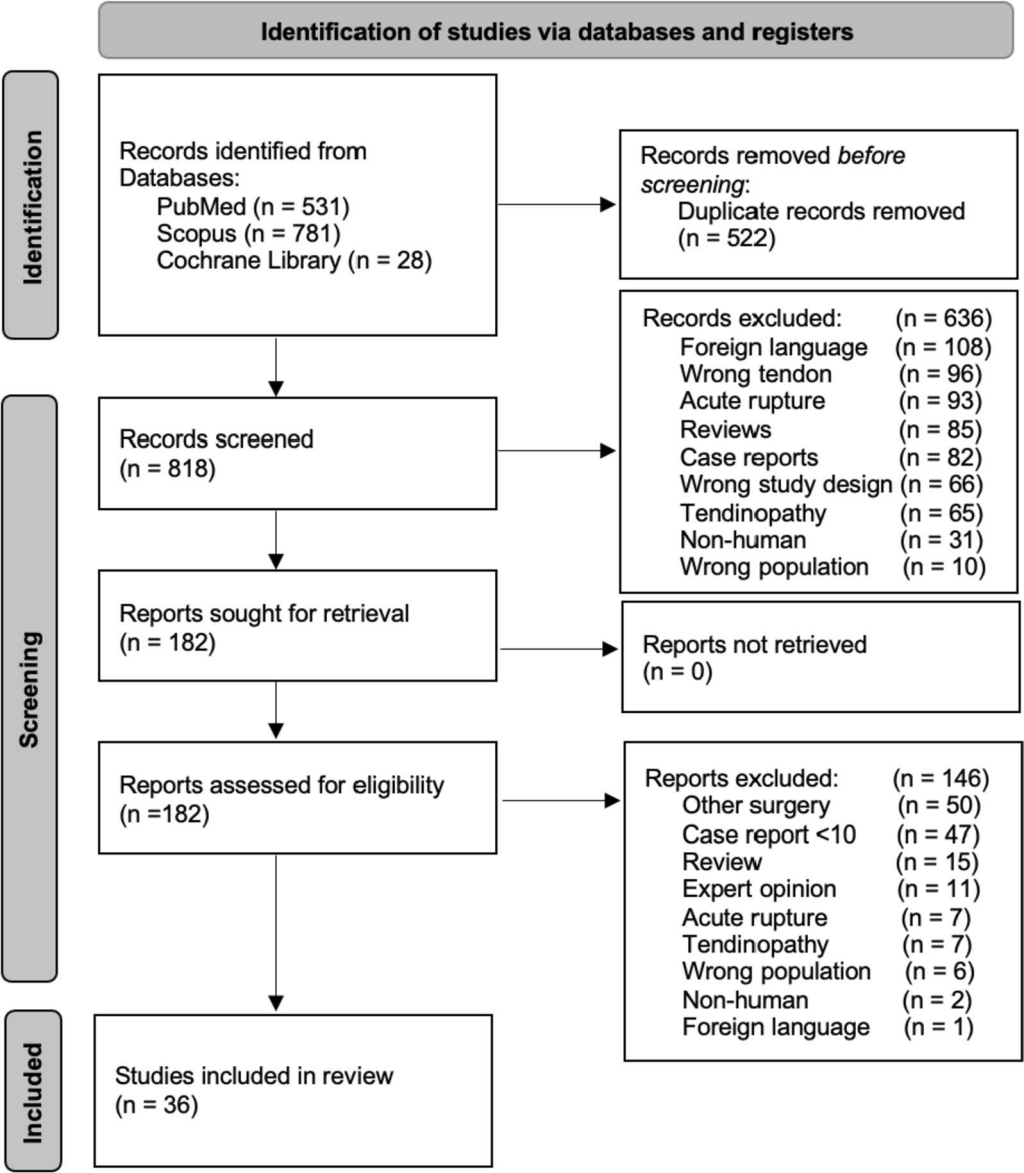
PRISMA-flowchart for the inclusion of studies, The PRISMA 2020 statement [54]

After the full-text filtering, 86 studies remained. Out of these, 36 individual studies used gastrocnemius aponeurosis flaps or semitendinosus tendon grafts to treat chronic Achilles tendon ruptures and were included in the review. The inclusion and exclusion process are illustrated in Fig. 1.

### Quality assessment

To assess the methodological quality and risk of bias for the included studies the validated Methodological Index for Non-Randomized Studies (MINORS). MINORS is a quality assessment tool for systematic reviews first described by Slim et. al [62]. It is widely used and has a strong external validity. The studies included in this systematic review were all non-randomized and non-comparative studies. MINORS consists of twelve questions, where the last four questions are additional criteria in case of comparative studies. Each question can be scored from 0–2. A score of 0 meaning it is not reported in the study, a score of 1 meaning it is reported but inadequate, and a score of 2 meaning it is reported and adequate [62].

### Result extraction

Three reviewers extracted the data using a structured extraction protocol. The extracted data was surgical technique, number of patients, mean age, duration of follow-up, outcome measures, presented results. The results analysed were patient-reported outcomes, functional outcome measures and complication rates. Any disagreement was settled through discussion. Moreover, if any author were among the authors of the original study analysed, they did not perform any result extraction or quality assessment of that study.

## Results

### Study selection

All the included studies were case series (Level IV) that either used a gastrocnemius aponeurosis flap or semitendinosus tendon graft to treat chronic Achilles tendon ruptures. A semitendinosus tendon graft was used in 13 studies and a gastrocnemius aponeurosis flap in 21 studies. There were two studies that used both surgical techniques. In the study by Bai et al. [4] 11 patients were treated with a gastrocnemius aponeurosis flap and 15 were treated with a semitendinosus tendon graft. The study by Gedam et al. [15] used both augmentation techniques in all patients and was therefore not part of the analysis. The extracted data from the studies is presented in Table 3. The grand total number of patients included were 763. The study by Bąkowski et al. [5] included eight additional cadavers which were excluded from this systematic review.

### Quality assessment

The included studies MINORS-scores are shown in Table 2. The maximum points are 16 for non-randomized studies. The included studies generally had low quality according to MINORS, with a median of 8 out of 16 (range 2–13) for all studies.

**Table 2:** The points of each study according to the MINORS-score. The maximum score is 16

**Criteria**	1 A clearly stated aim	2 Inclusion of consecutive patients	3 Prospective collection of data	4 Endpoints appropriate to the aim of the study	5 Unbiased assessment of the study endpoint	6 Follow up period appropriate to the aim of the study	7 Loss to follow up less than 5%	8 Prospective calculation of study size	Finalized MINORS score
**Jain et al. 2020 **[22]	1	2	0	2	0	2	2	0	9/16
**Elgohary et al. 2016 **[13]	1	1	1	1	0	2	2	0	8/16
**Koh et al. 2019 **[26]	2	0	0	2	0	2	0	0	6/16
**Massoud 2017 **[44]	1	0	2	0	0	2	2	0	7/16
**Kaul et al. 2020 **[23]	2	1	0	1	0	2	2	0	8/16
**Guclu et al. 2016 **[18]	1	2	0	1	0	1	0	0	5/16
**Sadek et al. 2015 **[59]	2	0	2	2	1	2	2	0	11/16
**Seker et al. 2016 **[61]	2	2	0	2	0	1	0	0	7/16
**Khiami et al. 2013 **[25]	2	2	0	1	1	2	0	0	8/16
**Pavan Kumar et al. 2013 **[55]	2	2	0	1	0	2	2	0	9/16
**Ozan et al. 2017 **[52]	2	0	0	1	0	2	2	0	7/16
**Lins et al. 2013 **[33]	1	1	1	2	0	2	2	0	9/16
**El Shazly et al. 2014 **[11]	2	1	2	2	0	2	0	1	10/16
**Bąkowski et al. 2020 **[5]	1	0	0	1	0	2	2	2	8/16
**Maffulli et al. 2014 **[36]	2	2	2	2	0	2	2	0	12/16
**Song et al. 2018 **[63]	2	1	0	2	0	2	2	0	9/16
**Dumbre et al. 2014 **[10]	1	1	1	2	0	2	2	0	9/16
**Maffulli et al. 2018 **[38]	1	1	2	2	0	2	2	0	10/16
**Bansal et al. 2021 **[6]	2	1	2	2	0	1	2	0	10/16
**Gedam et al. 2016 **[15]	1	0	0	2	0	1	0	0	4/16
**Li et al. 2021 **[31]	1	0	0	2	0	2	2	0	7/16
**Bai et al. 2019 **[4]	1	1	0	2	0	2	0	0	6/16
**Maffuli et al. 2013 **[37]	2	1	2	2	1	2	2	0	12/16
**Nilsson Helander et al. 2008 **[47]	2	2	1	2	2	2	2	0	13/16
**El Shewy et al. 2009 **[12]	0	2	1	1	0	2	2	0	8/16
**Sarzaeem et al. 2012 **[60]	1	2	2	2	0	2	2	0	11/16
**Takao et al. 2003 **[65]	0	2	0	1	0	2	2	0	7/16
**Muliera et al. 2003 **[45]	1	0	0	1	0	2	2	0	6/16
**Lin et al. 2019 **[32]	1	2	0	2	0	2	2	0	9/16
**Werken et al. 1983 **[67]	0	0	1	0	0	0	1	0	2/16
**Gunaratne et al. 2021 **[19]	1	2	0	1	0	2	0	0	6/16
**Nilsson et al. 2022 **[46]	2	1	0	2	0	2	2	0	9/16
**Nordenholm, Nilsson et al. 2022 **[49]	2	1	0	2	0	2	2	0	9/16
**Nordenholm, Senorski et al. 2022 **[50]	2	1	2	2	1	2	2	0	12/16
**Raju et al. 2022 **[57]	1	2	0	1	0	2	2	0	8/16
**Tsukada et al. 2022 **[69]	1	2	0	2	0	2	0	0	7/16

### Result extraction

A summary of the results of each individual study is shown in Table 3. The patients treated with gastrocnemius aponeurosis flap had a mean age of 44.5 years and were followed-up for a mean time period of 40 months. The patients treated with semitendinosus tendon grafts had a mean age and follow-up time of 44 years and 28 months, respectively. The outcome measures used in the studies were patient-reported outcome measures (ATRS [48], VAS [21], VISA-A [58], FADI [61], Tegner scale [63], and SF-36 [26]) mixed scores (AOFAS [64], Leppilahti [29, 64], Hooker [52], Arner-Lindholm [3], Rupp-score [24] and Holz-scale [12]) and clinical tests (calf circumference [64], range of motion (ROM), muscle strength/isokinetic testing/heel-rise tests, ultrasonography and Manual Muscle Testing (MMT) [10]). The most used outcome measure was AOFAS with a total of 19 unique studies. Some outcome measures were only used once: including the Holz scale, Tegner scale, FADI, Hooker, MMT, Arner-Lindholm, Rupp-score and SF-36.

**Table 3:** The included studies, number of patients, age, follow-up time, outcome measures, results and complications

First author and publication year [reference]	Repair method	Number of patients	Mean patient age	Follow-up time	Outcome measure	Outcome results	Complications (quantity)
**Jain et al. 2020 **[22]	FHL + G-flap	15	43.5 years	19.07 months (13–24)	AOFAS, ATRS	AOFAS score: 98.4 SD 2.03 (94–100), preop: 72.07 SD 8.29 (62–83) ATRS score: 98 SD 1.85 (94–100), preop: 61.73 SD 8.16 (52–70)	No re-ruptures. Serous discharge and delayed wound healing (2), wound infection (1)
**Elgohary et al. 2016 **[13]	FHL + G-flap	19	47 years	29 months (13–52)	AOFAS	AOFAS score: at 6 months 88.4 (72–96), at 12 months and at last follow-up 94 (76–100), preop: 65 (52–72). Mean time for return to work was 90 days (42–120), and to sport activity (4 patients) it was 147.5 days (90–210). All patients had calf muscle hypotrophy with a mean difference of 0.6 cm in calf circumference (0.3–1.8). All patients were able to perform a single leg heel-rise at the last follow-up	No re-ruptures. Deep infection (1). Skin sloughing, needing a skin graft (1). Hypertrophic scar (1). Superficial infection (3)
**Koh et al. 2019 **[26]	FHL + G-flap	49	60 years	At least 12 months	AOFAS, VAS, SF-36	AOFAS score: at 3 months 76 (SD 22), at 6 months 83 (SD 18), at 12 months 95 (SD 10), preop: 52 (SD 19) VAS score: at 12 months 0, preop: 5 SF-36: Physical function, physical limitation, bodily pain, and social function improved significantly after surgery, physical component score also improved significantly at 12 months	No re-ruptures. Stitch abscess (1). Wound dehiscence (1)
**Massoud 2017 **[44]	G-flap	15	25 years	5 years (3–8)	Calf circumference, Range of motion	Calf circumference was equal to the uninjured side in 12 patients. Three patients had calf muscle hypotrophy, which averaged 1.3 cm in difference to the healthy side (range 1–2) Active ankle motion range was equal to the contralateral in 14 patients	No re-ruptures. Superficial wound infection (3). Deep infection (1)
**Kaul et al. 2020 **[23]	G-flap	16	Not reported	At least 12 months	LEPPILAHTI	Leppilahti score: 12 months postop: 75% (12) had excellent result, 19% (3) had good result, and 6% (1) had fair result	No re-ruptures. Superficial surgical site infections (2). Delayed wound healing (1)
**Guclu et al. 2016 **[18]	G-flap	17	33 years	16 years (13–18)	AOFAS	AOFAS score: 95 (SD 3), preop: 64 (SD 4). Mean calf circumference difference: 3.4 cm (1–6). Mean 30 degrees plantar flexion and 120 degrees plantar flexion peak torques: 89 and 45 Nm. Mean deficiency in 30 degrees and 120 degrees: 16% and 17%	No re-ruptures. Superficial wound infection (2)
**Sadek et al. 2015 **[59]	G-flap	18	40.7 years	21.8 mosnths	AOFAS, calf circumference, Range of motion	AOFAS score: at 3 months 86.8 (82–99), at 6 months 94.6 (89–100), at 12 months 94.9 (89–100). Preop: 62.2 (40–85). ROM (plantar flexion): mean 43 degrees, healthy side was 45.8. Preop: 25.2 ROM (dorsiflexion): 19.4. Preop 21.2, healthy side 22.3 Calf hypotrophy: 3.3 (1–6) cm	No re-ruptures. Delayed wound-healing (2). Superficial wound infection (1)
**Seker et al. 2016 **[61]	G-flap	21	32.1 years	145.3 months	AOFAS, Range of motion, calf circumference, heel-raise, VAS, FADI. Peak torque	AOFAS score: 98.5 (90–100). FADI score: 98.9 (96.2–100). VAS score: 0. Calf circumference: 36.2 (30–40) compared with 37.2 (32–41) in healthy side. Ankle dorsiflexion: 18 (10–20) degrees compared with healthy side 19 (15–20) degrees. Plantar flexion: 30 (20–40) for both sides. Mean time for return to daily activities: 11.1 (8–16) weeks. Single heel rise was attainable after a mean of 14.1 (9–20) months after surgery. The mean plantar flexion peak torque for 30 and 120 degrees were 82 (70–142) and 42 (39–69) compared with the healthy side of 96 (70–145) and 43 (40–75). Median peak force deficiency was 6 (0–21) % at 30 degrees and 4.8 (0–12.5) % at 120 degrees. Mean dorsiflexion peak torque for 30 degrees: 51 (45–60) Nm compared with 55 (44–67) Nm on the healthy side. And the same bilaterally for 120 degrees with 39 (35–54) on the operative side and (31–55) on the healthy side	No re-ruptures. Superficial soft tissue infection (1)
**Khiami et al. 2013 **[25]	G-flap	23	52.1 years	24.5 months (12–43)	AOFAS	AOFAS score: 96.1 SD 6.8 (range, 79–100). Preop: 63.6 SD 11.5. The group without abnormal MRI signal had a mean AOFAS score of 98.3 SD 3.9, and those with abnormality 92.5 SD 10.5. Twelve patients resumed leisure sports at their previous level by a mean ± SD (range) 9.4 SD 2 months (7–12); three competitive sportsmen resumed sport at their previous level by a mean 7.6 months. MRI performed at 1 year showed increased tendon volume without abnormality in 57% (8/14) of cases; 43% (6/14) showed abnormal images	No re-ruptures. Partially regressive sural nerve hypoesthesia (1). Aseptic superficial skin necrosis (1). Septic partial tendon necrosis (1)
**Pavan Kumar et al. 2013 **[55]	G-flap	78	No mean, between 38–66 years	12 months	LEPPILAHTI, ultrasonography	LEPPILAHTI: 62 patients had excellent results (90-100p), 8 had good results (75-89p), 4 had fair results (60-74p), 2 had poor results (< 60p) and 2 were lost to follow up after 1 year. Nearly all patients resumed work at 6 months post-op and had normal walking, and stair climbing as well as normal dorsiflexion	No re-ruptures. Scar hypertrophy (2). Deep infection (1), superficial infection (2). Delayed wound healing (3)
**Ozan et al. 2017 **[52]	G-flap	15	35.2 years	19.6 months	Range of motion, Hooker, Heel-rise	Mean calf hypotrophy was 1.2 cm (0–2.5). No patient had any limitations to daily living and the active and passive ankle ROM was "good". Mean time to work was 38 months. The mean time for patients to return to daily activity was 3.2 months (range, 2—5 months). All patients were able to perform singe-leg heel rises. Hooker scores were excellent for 11 patients and satisfactory for 4 patients	No re-ruptures. No infection, no complications reported
**Lins et al. 2013 **[33]	ST-graft	13	42.2 years	12 months	AOFAS, Gait-pattern, Range of motion, Calf circumference	AOFAS score: at 6 months postop: 68.5 (SD 18.7), at 12 months postop: 85.2 (SD 18.7). The control group walked faster. Their stance phase was also shorter than patients affected by rupture. The calf circumference at 6 months was 257 compared with 24.5 at 12 months. The ROM-values were lower for the injured ankle at both 6 and 12 months	No re-ruptures. No infections. Weakness at the donor site (1)
**El Shazly et al. 2014 **[11]	ST-graft	15	37.7 years	24 months	AOFAS, Isokinetic testing	AOFAS score: at 24 months postop: 90.8. Preop: 32.6. Isokinetic testing showed a non-significant deficit (< 10%) of the plantar flexors on the injured side	No infections. Temporary sural nerve injury (1)
**Bąkowski et al. 2020 **[5]	ST-graft	18	53 years	15.3 months (12–24)	ATRS, VAS, QOL questions, calf circumference	ATRS improved significantly (no data). Lower calf circumference (no data). VAS pain 1.0. VAS satisfaction 9.0. Median EQ-5D 80.0. Heel-rise endurance 10.5 vs 7.0. Isokinetic flexor peak torque better on non-injured side, 91.3 vs 111. Single hop showed no statistical difference between injured and uninjured side	No re-ruptures. Moderate donor site pain and weakness (1)
**Maffulli et al. 2014 **[36]	ST-graft	26	46 years	31.4 months	ATRS, calf circumference, isometric strength, heel-rises	ATRS score: Postop latest follow-up: 86 (78–95). Preop: 42 (29–55). Calf circumference: 37.9 cm on the injured side vs 42.8 on the healthy side. Isometric 357 J on injured side vs 408 on healthy side. All patients could perform 10 heel-rises	No re-ruptures. No infections nor nerve injuries. Persistent pain over the distal operating wound (1)
**Song et al. 2018 **[63]	ST-graft	36	36 years	53 months	AOFAS, ATRS, VISA-A, Tegner	AOFAS score: Postop at latest follow-up 100 (86–100). Preop: 50 (5–75). VISA-A: Postop at latest follow-up: 94 (52–100). Preop: 23 (5–59). ATRS score: Postop at latest follow-up: 99 (84–100). Preop: 22.5 (6–67). Tegner score: Postop at latest follow-up: 4 (3–9). Preop: 1 (0–3)	No re-ruptures. No infections or nerve injuries. Pain related to the operation wound (4)
**Dumbre et al. 2014 **[10]	ST-graft	35	47 years	30.7 months (20–42)	Weight bearing MMT	Weight-bearing MMT postop at latest follow-up: 4/5. Preop: 2/5. Non-weight-bearing MMT postop at latest follow-up: 5/5	No re-ruptures. No complications reported
**Maffulli et al. 2018 **[38]	ST-graft	21	44.8 years	35.4 months	ATRS, calf circumference, plantar flexion strength	ATRS score: Postop at latest follow-up: 89.4 SD 3.2. Preop: 50.4 SD 7.5. Calf circumference postop: 38.7 cm SD 3.6 cm on the injured side, compared with 39.3 cm SD 3.4 cm on the healthy side. Plantar flexion strength: 424.3 N SD 42.9 N on the injured side, compared with 488.0 N SD 44.8 N on the healthy side	No re-ruptures reported. No complications reported
**Bansal et al. 2021 **[6]	ST-graft	10	45.1 years	12 months	AOFAS	AOFAS score: Postop at latest follow-up: 80.4. Preop: 40.8. All except one patient were able to walk on tiptoes	No re-ruptures. Superficial wound infection (1)
**Gedam et al. 2016 **[15]	ST-graft + G-flap	14	45.6 years	30 months (12–78)	AOFAS, ATRS	AOFAS score: Postop at latest follow-up: 96.9 (90–100). Preop: 64.5 (35–79). ATRS score: Postop at latest follow-up: 91.4 (83–97). Preop: 49.4 (30–70)	No re-ruptures. No complications reported
**Li et al. 2020 **[31]	ST-graft	26	44.2 years	15 months (12–18)	AOFAS, Plantar flexion strength, VISA-A scale, VAS	AOFAS score: Postop at latest follow-up: 91.3 SD 6.5. Preop: 44.9 SD 2.1. VISA-A: Postop at latest follow-up: 84.1 SD 3.9. Preop: 49.1 SD 3.2. VAS: Postop at latest follow-up 1.08 SD 0.3. Preop: 5.97 SD 0.7. Plantar flexion strength: Postop at latest follow-up: 133.7 N SD 17.5 N in injured side, compared with 141.5N SD 11.8 N in healthy side. All 26 was able to perform single leg heel-rises	No re-ruptures. No complications reported
**Bai et al. 2019 **[4]	ST-graft + G-flap	Total: 26, G-flaps: 11, ST-grafts: 15	36.7 years	12 months	AOFAS, Leppilahti score	ST-grafts: AOFAS score: Postop at latest follow-up: 93.5 SD 2.5. No preop measurement. Leppilahti score: Postop at latest follow-up: 95.1 SD 3.1. No preop measurement Gastrocnemius-flaps: AOFAS score: Postop at latest follow-up: 92.6 SD 3.0. No preop measurement. Leppilahti score: Postop at latest follow-up: 94.7 SD 3.1. No preop measurement	No re-ruptures. Nerve injury (1 in ST-graft group). Infection (2 in G-flap group), DVT (2 in the Gastrocnemius-flap group)
**Maffulli et al. 2013 **[37]	ST-graft	26	42 years	8.2 years (7–10)	ATRS, calf circumference, isometric strength	ATRS at latest follow-up was 88. Lower isometric strength in injured side vs healthy side. Calf circumference 39.7 (SD 7.1) cm on the injured side vs 41.5 (SD 6.6) cm on the healthy side	No re-ruptures. Superficial wound infection (2). Wound adhesion (1)
**Nilsson Helander et al. 2008 **[47]	G-flap	28	46 years	29 months (12–117)	ATRS, questionnaire for symptoms, physical activity, and satisfaction	ATRS score: Postop at latest follow-up: 83 (24–100). 16 patients were satisfied with the final outcome	No re-ruptures. Deep infection (1), wound closure complication (2), DVT (2)
**El Shewy et al. 2009 **[12]	G-flap	11	34.3 years	7.45 years (6–9)	AOFAS, Holz scale, Range of motion, calf circumference	Holz scale: preop 10 poor, 1 fair. Postop 11 good. AOFAS: Preop 42.27 (39–46). Postop 98.9 (88–100). Ankle ROM (mean + SD): plantar flexion preop 20.5 SD 2.70. Postop 49.6 SD 1.5. Dorsiflexion preop 11.4 SD 3.23. Postop 17.7 SD 2.6. Calf (cm): Circumference (mean + SD)) Preop 31.6 SD 0.9. Postop 34.4 SD 0.89. Wasting (mean + SD) Preop 1.6 SD 0.4. Postop 0.7 SD 0.2	No re-ruptures. Small wound gaping (3), superficial wound infection (2)
**Sarzaeem et al. 2012 **[60]	ST-graft	11	30 years	25 Months	AOFAS, ATRS, Range of motion, Calf circumference	ATRS: Preop: 32 SD 6 (24–39). Postop: 89 SD 4 (82–95) AOFAS: Preop: 70 SD 5 (61–78). Postop: 92 SD 5 (83–97) Circumference: Injured side: 36 SD 3 (30–42) cm. Healthy side: 38 SD 4 (33–45) cm ROM: Plantar flexion: Injured side: 36 SD 8 (22–50) degrees. Healthy side: 39 SD 6 (30–50) degrees. Dorsiflexion: Injured side: 13 SD 4 (5–20) degrees. Healthy side: 17 SD 4 (10–25) degrees	No re-ruptures. Superficial infection (2), DVT (1)
**Takao et al. 2003 **[65]	G-flap	10	51 years	75.1 months (26–192)	Calf circumference, Range of motion	AOFAS—Preop: 72.6 SD 5.3 (68–82). Postop: 98.1 SD 2.5 (94–100) Cybex—30 degrees, preop: Torque effect ranged from 8 to 68% at the low setting and from 19 to 33% at the high setting. Postop, the torque ranged from –9% to 17% at the low setting and from –13% to 23% at high setting	Not reported
**Mulier et al. 2003 **[45]	G-flap with and without FHL	19	37 years	18 months	Leppilahti score, stiffness, muscle weakness, Range of motion, isokinetic calf muscle strength, CYBEX	Leppilahti: Postop Gastrocnemius -flap: Fair, 62 (48–78). Postop Gastrocnemius-flap + FHL: Good, 77 (67–89) Cybex: G-flap: 23% (5–45) decrease in power and strength. Gastrocnemius -flap + FHL: 14% (5–35) decrease in power and strength ROM: Gastrocnemius -flap: dorsiflexion 9 degrees (-5–20), plantar flexion 33 degrees (20–45). Gastrocnemius -flap + FLL: dorsiflexion 13 degrees (-5–25), plantar flexion 36 (20–45)	Re-rupture (1). Deep infection (1 in g-flap only), DVT (1 in each group, 2 total), delayed wound healing (3 in g-flap, 4 in Gastrocnemius-flap + FHL)
**Lin et al. 2019 **[32]	G-flap	20	38.5 years	32.8 months (12–68)	AOFAS, ATRS	ATRS: Preop: 39.6 SD 14.2 (20–72). Postop: 94.1 SD 4.9 (86–100) AOFAS: Preop: 59.3 SD 12.3 (40–75). Postop: 96.6 SD 3.8 (90–100)	No re-ruptures. Superficial infection (1)
**Werken et al. 1983 **[67]	G-flap	10	51 years	At least 2 years	Calf circumference	Calf Circumference difference between the healthy and injured sided was 1–3.5 cm	No re-ruptures. Wound infection (2)
**Gunaratne et al. 2021 **[19]	G-flap	13	56 years	12 months	ATRS	Mean ATRS postop 72	Not reported
**Nilsson et al. 2022 **[46]	ST-graft	22	60 years	12 months	ATRS. ATRA. Heel-rise height. Heel-rise reps. Calf circumference. Ultrasonography. Concentric power. Heel-rise work	ATRS 76 (45–99). 89% were able to perform a single-leg heel-rise on the injured side. Tendon length 2.8 cm longer on the injured side compared with the non-injured and calf circumference 1.5 cm lower. ATRA was 60 (15) vs 49.5 (6) on the non-injured side. The same numbers for heel-rise height were 5.5 (5.75) vs 9.0 (2.75) and for heel-rise reps 11 (18) vs 26 (14)	No re-ruptures. 2 superficial wound infections and 1 sural nerve injury
**Nordenholm, Nilsson et al. 2022 **[49]	G-flap	22	61 years	12 months	ATRS. PAS. FAOS. Heel-rise height. Heel-rise reps. Calf circumference. Ultrasonography. CMJ. Heel-rise work. Hopping ratio. ATRA. Dorsiflexion range	Mean (SD) ATRS 62 (26) and mean (SD) PAS 3.5 (1.1). The patients performed less well in the heel-rise test on the injured side compared with the healthy side with a median (IQR) of 20 (10) vs 24 (12) in heel-rise repetitions (*p* = 0.004), 8 (7) vs 10 (8) cm in heel-rise height (*p* < 0.001), 872 (1740) vs 1590 (2145) joule in total heel-rise work (*p* = 0.001) and 0.37 vs 0.48 in hopping ratio (*p* = 0.005). Patients with chronic Achilles tendon rupture exhibited an elongation of the injured Achilles tendon with median (IQR) ATRA of 55° (3) compared with 50° (9) (*p* < 0.001) and a median of 22.4 (2.9) cm compared with 20.5 (2.0) cm measured by ultrasound (*p* = 0.06). Calf circumference was smaller on the injured side with a median (IQR) of 37 (4) compared with 38 (4) cm (*p* = 001)	No re-ruptures. 1 superficial wound infection
**Nordenholm, Senorski et al. 2022 **[50]	G-flap	23	61 years	12 months	Gait-analysis	Significantly reduced step width (0.01 m (*p* = 0.014)), increased speed (− 0.12 m/s (*p* = 0.013)), stride length (− 0.12 m (*p* = 0.002)), ankle moment (− 0.64 Nm/kg (*p* < 0.001)) ankle power (− 1.38 W/kg (*p* < 0.001)) and knee power (− 0.36 W/kg (*p* = 0.003)) compared with the preoperative status	Not reported
**Raju et al. 2022 **[57]	G-flap	12	47 years	34 months	Calf diameter. Dorsiflexion. Heel-rises. VAS. Rupp-score	Calf-diameter increased 2.2 cm. Dorsiflexion increased 10 degrees. Rupp-score with 5 excellent and 7 good	No re-ruptures or complications
**Tsukada et al. 2022 **[69]	ST-graft	10	52 years	35 months	AOFAS. ATRS. VAS. Months until the patient could perform 20 single-leg heel-rises	AOFAS increased significantly from 64.2 SD 5.6 (range 58–72) points preoperatively to 95.0 SD 5.3 (range 90–100) points at the final follow-up (*P* < 0.001), as did mean ATRS, from 29.8 SD 4.4 (range 22–35) points to 86.2 SD 7.7 (range 70–94), respectively (*P* < 0.001). Mean time between surgery and ability to perform 20 continuous double-leg heel rises of the operated foot was 13.5 SD 3.4 (range 10–18) weeks	Re-ruptures not reported. 1 sural nerve injury

The mean (SD; n) postoperative ATRS was 83 (14; 6 studies) and the mean (SD; n) AOFAS was 96 (1.7; 12 studies) for patients treated with a gastrocnemius aponeurosis flap. In comparison, the mean (SD; n) ATRS and AOFAS for semitendinosus tendon grafts were similar with scores of ATRS 88 (6.9; 7 studies) and AOFAS 92 (5.6; 9 studies). However, patients treated with semitendinosus tendon grafts had lower mean (SD; n) preoperative values with ATRS 38 (11.3; 6 studies) vs. 50 (11.1; 3 studies) and AOFAS 51 (13.3; 7 studies) vs. 62 (9.0; 10 studies).

A comparison of the complications between gastrocnemius aponeurosis flaps and semitendinosus tendon grafts can be found in Table 4. The most common complication found was superficial wound infection with a total of 27 patients (3.4%), 22 (4.6%) of which were treated with gastrocnemius aponeurosis flaps and 5 (1.5%) with semitendinosus tendon grafts. In general, patients treated with gastrocnemius aponeurosis flap had more complications than patients treated with semitendinosus tendon grafts, mainly due to wound healing problems. However, patients treated with semitendinosus graft were more prone to sural nerve injury due to a smaller surgical incision. Only one re-rupture occurred in the total group of 763 patients. That patient was treated with a gastrocnemius flap.

**Table 4:** Overview of the complications for semitendinosus tendon grafts (ST-grafts) and gastrocnemius flaps (G-flaps)

Overview of the complications			
Complications	**ST-grafts n (%)**	**G-flaps n (%)**	**Total n (%)**
Wound infection	5 (1.7)	22 (4.6)	27 (3.5)
Delayed wound healing	2 (0.7)	16 (3.3)	18 (2.4)
Deep Vein Thrombosis	1 (0.3)	6 (1.3)	7 (0.9)
Wound dehiscence	1 (0.3)	6 (1.3)	7 (0.9)
Deep infection	-	5 (1.0)	5 (0.7)
Persistent pain from the operation wound	5 (1.7)	-	5 (0.7)
Hypertrophic scar	-	3 (0.6)	3 (0.4)
Weakness from the donor site	2 (0.7)	-	2 (0.3)
Sural Nerve injury	4 (1.3)	-	4 (0.5)
Re-rupture	-	1 (0.2)	1 (0.1)
Persistent pain from the donor site	1 (0.3)	-	1 (0.1)
Stitch abscess	-	1 (0.2)	1 (0.1)
Sural nerve hypoesthesia	-	1 (0.2)	1 (0.1)
Aseptic superficial skin necrosis	-	1 (0.2)	1 (0.1)
Septic partial tendon necrosis	-	1 (0.2)	1 (0.1)

## Discussion

The most important finding of this systematic review was that both gastrocnemius aponeurosis flap and semitendinosus graft reconstructions were found to be effective in treating chronic Achilles tendon ruptures with similar favourable patient-reported outcome scores and performances in functional tests. Only one re-rupture was reported (0.12%) in the patient group treated with a gastrocnemius aponeurosis flap and no patient in the group treated with a semitendinosus graft. However, studies of higher quality are needed to fully determine the optimal way of treating chronic Achilles tendon ruptures. All the included studies were case series without matched control groups. Additionally, the articles used a variety of different outcome measures, which limited the comparisons between studies. Lastly, even though the gastrocnemius aponeurosis flaps, and semitendinosus tendon grafts are presented as distinctive groups, both groups were heterogenic with different interpretations of the techniques.

In prior systematic reviews by Apinun et al. [2] and Hadi et al. [20], similar results have been shown with good functional patient-reported outcomes, and low re-rupture rates. The review performed by Hadi et al. identified 35 individual studies in 2013 whereas this systematic review identified a total of 86 individual publications on the same subject in 2022. The heterogenicity of outcome measures and surgical techniques, and the retrospective nature of limited cohort sizes remain. This meant that quantitative meta-analysis was deemed inappropriate. Studies including fewer than 10 patients were excluded from this systematic review. Most of these studies were case reports with one to two patients using no outcomes measures. Therefore, the exclusion of case reports did not result in any substantial data loss.

The result of the included studies indicate that patients treated with a gastrocnemius flap are more prone to complications than patients treated with semitendinosus tendon graft. Due to the heterogenicity, no significant difference could be determined, however. Depending on the surgical technique a different pattern of complications occurred. Semitendinosus tendon grafts uses an autologous transplantation with risk of complications related to the hamstring donor site or sural nerve injury due to the location and the smaller size of the surgical incisions. Gastrocnemius flaps uses a turn-down flap or a free-flap from the aponeurosis with larger surgical wounds leading to an increased risk of infections and wound healing problems [42].

The MINORS assessment generally resulted in a low-quality with scores with a median of 8 out of 16. Moreover, the studies included in this review frequently used AOFAS as their main patient-reported outcome. It is known that this outcome measure is not validated for Achilles tendon ruptures. Instead, a patient-reported outcome such as ATRS could be used. This patient-reported outcome measure is also not validated for chronic Achilles tendon ruptures, but it is validated for acute Achilles tendon ruptures. In the future, research of chronic Achilles tendon ruptures would benefit from a patient-reported outcome measure that is validated for chronic ruptures, as that would allow for a clearer comparison between operating methods and outcome.

The exclusions based on the number of patients and the language of the studies might have affected the results of this review. This review excluded all non-English studies and all studies with less than 10 patients. The exclusion criteria removed 108 studies due to language and 47 studies due to a small cohort size. The exclusion of studies in non-English language facilitated data extraction by avoiding outcome heterogeneity between studies, thus improving quality. The number of articles excluded based on sample size were large in number but were usually singular case reports without any outcome measures. Similarly, varying outcomes measures may have made comparisons between studies even harder.

Following this systematic review, the authors recommendation is to individualise the treatment of chronic Achilles tendon ruptures depending on factors such as functional demands, comorbidities, tendon gap size, and the general experience of the orthopaedic surgeon treating the patient. The use of a gastrocnemius aponeurosis flap in tendon ruptures with a gap that is less than 5 cm is regarded as efficient [12, 47]. In tendon ruptures with larger defects (> 5 cm) a semitendinosus tendon graft will bridge the defect [37, 60]. Other alternatives include flexor hallucis longus graft [53, 56] and peroneus tendon graft [40].

## Conclusion

In conclusion, surgical reconstruction with both semitendinosus tendon grafts, and gastrocnemius aponeurosis flaps are considered effective in treating chronic Achilles tendon ruptures with good patient-reported outcomes and few re-ruptures. The complication profiles are different between the two techniques with more postoperative infections and wound healing complications in patients treated with a gastrocnemius aponeurosis flap and more sural nerve injuries and donor site weakness in patients treated with a semitendinosus graft. There is a continued need for more prospective randomized controlled trials and a need for an established outcome measure for chronic Achilles tendon ruptures to fully evaluate the effectiveness of different reconstructive techniques in the treatment of chronic Achilles tendon ruptures.

## Data Availability

The datasets used and/or analysed during the current study available from the corresponding author on reasonable request.
